# Autophagy Defect Is Associated with Low Glucose-Induced Apoptosis in 661W Photoreceptor Cells

**DOI:** 10.1371/journal.pone.0074162

**Published:** 2013-09-16

**Authors:** Delphine Balmer, Martine Emery, Pénélope Andreux, Johan Auwerx, Vanessa Ginet, Julien Puyal, Daniel F. Schorderet, Raphaël Roduit

**Affiliations:** 1 IRO-Institute for Research in Ophthalmology, Sion, Switzerland; 2 Faculty of Life Sciences, Ecole Polytechnique Fédérale de Lausanne, Lausanne, Switzerland; 3 Department of Ophthalmology, University of Lausanne, Lausanne, Switzerland; 4 Département des Neurosciences Fondamentales, Université de Lausanne, Lausanne, Switzerland; University of Bremen, Germany

## Abstract

Glucose is an important metabolic substrate of the retina and diabetic patients have to maintain a strict normoglycemia to avoid diabetes secondary effects, including cardiovascular disease, nephropathy, neuropathy and retinopathy. Others and we recently demonstrated the potential role of hypoglycemia in diabetic retinopathy. We showed acute hypoglycemia to induce retinal cell death both *in vivo* during an hyperinsulinemic/hypoglycemic clamp and *in vitro* in 661W photoreceptor cells cultured at low glucose concentration. In the present study, we showed low glucose to induce a decrease of BCL2 and BCL-XL anti-apoptotic proteins expression, leading to an increase of free pro-apoptotic BAX. In parallel, we showed that, in retinal cells, low glucose-induced apoptosis is involved in the process of autophagosomes formation through the AMPK/RAPTOR/mTOR pathway. Moreover, the decrease of LAMP2a expression led to a defect in the autophagosome/lysosome fusion process. Specific inhibition of autophagy, either by 3-methyladenine or by down-regulation of ATG5 or ATG7 proteins expression, increased caspase 3 activation and 661W cell death. We show that low glucose modifies the delicate equilibrium between apoptosis and autophagy. Cells struggled against low nutrient condition-induced apoptosis by starting an autophagic process, which led to cell death when inhibited. We conclude that autophagy defect is associated with low glucose-induced 661W cells death that could play a role in diabetic retinopathy. These results could modify the way of addressing negative effects of hypoglycemia. Short-term modulation of autophagy could be envisioned to treat diabetic patients in order to avoid secondary complications of the disease.

## Introduction

Neural tissues, including retina, are totally dependent on glucose for normal metabolic activity. In both type I and II diabetes, normalization of blood glucose concentration is an important issue to avoid secondary long-term microvascular complications, including nephropathy, cardiovascular diseases, neuropathy and retinopathy [1]. We recently showed that not only hyperglycemia, but also hypoglycemia, could be detrimental for the retina [2]. Indeed, both *in vivo* short-term hypoglycemia, induced by a 5-hour hyperinsulinemic clamp, or the *in vitro* model of 661W photoreceptor cells cultured at low glucose condition, led to retinal cell death via an activation of the caspase 3 pathway and a decrease of glutathione (GSH) content. This report highlighted new pathways in the low glucose-induced cell death and confirmed results obtained by Luo *et al.* showing that conditions of low glucose reduced viability of all retinal cell types in a mixed primary cell culture [3] and by Zeevalk and Nicklas demonstrating the sensitivity of isolated chick retinas to *in vitro* aglycemic conditions [4]. Recently, Umino *et al.* showed that chronic moderate hypoglycemia in mouse led to loss of vision and eventual retinal degeneration [5], while Punzo *et al.* suggested that cones death in retinitis pigmentosa could be, at least in part, the result of starvation via the insulin/mTOR pathway [6]. A recent publication, showing a decrease of central retinal function in human during acute hypoglycemia, strengthened the importance of glycemic excursion in patients [7].

Programmed cell death, also called apoptosis, has been analyzed in various cell systems, stimulated by multiple stimuli. This process is necessary for the removal of damaged cells. Proteins of the B-cell lymphoma 2 (BCL2) family are well-described key regulators involved in this mechanism and regulate caspase activation; they are divided in pro-apoptotic and anti-apoptotic family proteins, which together take the “life-or-death decision” for the cell (for review see refs. [8]–[10]). Other cell death or survival programs, including autophagy, have also been described to play an important role in cellular homeostasis by eliminating and/or replacing non-functional organelles and proteins. During the development in chicken, autophagy occurs to eliminate cell death and establish a structured and functional retina [11]. Several studies showed an activation of autophagy in bright-light rat exposure [12], in light-damaged mouse retina and in 661W photoreceptor cells exposed to oxidative stress [13]. Although autophagy is implicated in many neurodegenerative processes, it has been described as a survival answer to various stress conditions. In low nutrient conditions (starvation, hypoxia), activation of autophagy leads to sufficient energy production to maintain vital functions. A similar process, called sporulation, occurs in nutrient-starved Sacharomyces cerevisiae [14]. During autophagy, the microtubule-associated protein 1 light chain 3 (LC3-I) is modified by the addition of a phosphatidyl-ethanolamine group (LC3-II) that allows integration of the protein to autophagosome membranes. Sequestosome 1 (p62/SQSTM1) is also involved in autophagy and recruited to the autophagosomal membrane through interaction with LC3 [15]. Absence of, or defective autophagy leads to an increase of p62 expression [16], while autophagy-induced p62 degradation suppresses tumorigenesis [17]. Both apoptotic and autophagic machineries share common pathways with proteins, several of them playing a dual role, in particular proteins of the BCL2 family that control apoptosis as well as autophagy [18], [19]. Moreover, both pathways may co-exist in the same cell [13]. The mammalian target of Rapamycin (mTOR) is another key player in autophagy; in normal physiological conditions mTOR inhibits autophagy, while in poor nutrient conditions, inactivation of mTOR complex leads to autophagy induction [20]. Recent publications describe the low-nutrient activation of autophagy via the adenosine monophosphate-activated protein kinase (AMPK)-mTOR pathway in mouse embryonic fibroblast [21] and endothelial cells [22]. The AMPK is a nutrient sensor activated by phosphorylation in poor-nutrient conditions, while in “normal” or rich nutrient conditions the kinase is inactivated (for review see refs. [21], [23], [24]). Depending on the stimulus or the cell system, the activated-autophagy could show opposing facets, either detrimental or protective. Moreover an emerging role of autophagy in diabetes mellitus has been recently proposed [25], [26]. We therefore analyzed the specific role of low glucose-induced apoptosis and autophagy in 661W photoreceptor cells.

To further decipher the mechanisms involved in the low glucose-induced apoptotic process, we treated 661W cells and retinal explants to low glucose and analyzed the signaling events that occurred. We showed that low glucose induced apoptosis through the BCL2/BAX pathway and autophagy through the AMPK/RAPTOR/mTOR pathway. At the same time, low glucose induced a defect of autophagosome/lysosome fusion via a decrease of the lysosome-associated membrane protein type 2a (LAMP2a) protein expression. The drastic decrease of fusion led to an accumulation of LC3-II and p62 proteins, two markers of autophagosomes accumulation. Autophagy inhibition, either by 3-methyladenine (3-MA) or by specific knock-down of either ATG5 or ATG7 caused a decrease of low glucose-induced LC3-II accumulation and sensitized cells to low glucose by increasing caspase 3 activity and cell death. The fine balance between apoptosis and autophagy may be critical for 661W cells survival in low energy conditions. Modulation of both these pathways might be important to avoid complication of diabetes, especially diabetic retinopathy.

## Methods

### Mouse Line

This study adhered to the Association for Research in Vision and Ophthalmology (ARVO) statement for the use of animals in ophthalmic and vision research and was approved by the Veterinary service of the State of Valais, Switzerland (permit ID: VS22). Animals were kept in a 12-h light/12-h dark cycle with unlimited access to food and water. Mice were injected with pentobarbital (150 mg/kg) prior eye enucleation and retina isolation.

### Cell Culture Conditions

Retinal explants were isolated from 2 month-old mice and cultured on a nitrocellulose filter (Millipore AG, Millicell #PIHA03050) in Dulbecco’s modified Eagle’s medium (DMEM) supplemented with 10% heat-inactivated fetal bovin serum (Lonza, #DE14-801F) and 1% penicillin/streptomycin, for one week prior to the experiment. 661W photoreceptor cells, kindly given by Dr Muayyad Al-Ubaidi, were cultured in complete DMEM medium as described previously [27]. Then, retinal explants and 661W cells were cultured in glucose-free DMEM supplemented with various glucose concentrations for diverse periods of time. Lysosomal inhibitors were added 4 h (10 µg/ml Pepstatine/E64; 75 µM Chloroquine; 100 nM Bafilomycin) before analysis and autophagy inhibitor 3-methyladenine (3-MA) was added at 600 µM for diverse periods of time.

### Lentiviral Vectors and Virus Production

Recombinant lentiviruses delivering shRNAs against Atg specific for mouse genes from TRC (the RNAi consortium) library in pLKO lentiviral vectors were used as follows: a combination of TRCN0000099431 and TRCN0000099434 for Atg5 (GenBankTM NM_053069) and a combination of TRCN0000092163 and TRCN0000092166 for Atg7 (GenBankTM NM_028835) (Openbiosystems).

We generated lentiviruses expressing either the mRFP-GFP-LC3 or the GFP-LC3 under the control of doxycyclin dependent promoter. Therefore, we extracted mRFP-GFP-LC3 (NheI/EcoRI) and GFP-LC3 (AgeI/EcoRI) from ptfLC3 (Addgene, #21074) and subcloned both fragments in the pENTR4 vector (Invitrogen, #A10465). We then recombined both pENTR4-mRFP-GFP-LC3 and pENTR-GFP-LC3 vectors with the doxycyclin-inducible lentiviral destination vector pSin-TRE-GW-3xHa-puroR to generate pSin-TRE-GW-3xHa-puroR-mRFP-GFP-LC3 (mRFP-GFP-LC3) and pSin-TRE-GW-3xHa-puroR-GFP-LC3 (GFP-LC3). Self-inactivating lentiviral vectors were produced as described previously [28].

### Generation of Stable Cell Lines

To establish 661W cell lines stably expressing specific shRNAs against ATG5 or ATG7 and mRFP-GFP-LC3 or GFP-LC3 chimeric proteins, 6×10^6^ 661W cells were seeded in T25 cell culture flask and transduced with 50 ng of p24/ml culture medium for each vector. In parallel, 661W cells were transduced with a pLKO empty lentiviral vector as control. After 48 h, the transduced cells were trypsinized and selected with 5 µg/ml Puromycin (Santa Cruz, #sc-108071A). Expression of fluorescent proteins to study fusion process were assessed by adding 1 µg/ml doxycyclin (Sigma, #D9291) 2 days before experiments.

### Terminal dUTP Nick End-Labeling (TUNEL) of Fragmented DNA

*In situ* cell death detection was performed after diverse periods of low glucose exposure, by TUNEL technology as described by the manufacturer (Roche Applied Science, #12156792910) and detailed in Emery *et al.* [2]. Quantification of TUNEL positive cells were performed by counting an average of 200 cells in 4 to 5 different fields for each condition.

### Western Blot Analysis and Immunohistochemistry

Thirty micrograms of proteins were electrically transferred to PVDF filters and incubated with antibodies from Santa Cruz Biotechnology: anti–BAX (#sc-493), anti–BCL2 (#sc-73829), anti–APG7 (#sc-33211); from Cell Signaling Technology Inc.: anti–mTOR (#2983), anti–P-mTOR (#2971), anti–AMPK (#2532), anti–P-AMPK (#2535), anti–P-RAPTOR (#2083), cleaved-CASPASE 3 (#9661), anti–LC3 (#2775), anti–BCL-XL (#2764); from Abcam: anti-LAMP2 (#ab18528); from Sigma: anti-ACTIN (#A5441), anti–TUBULIN (#T6074), anti-p62/SQSTM1 (#P0067)). As secondary antibody, we used anti-rabbit HRP (Sigma, #A5420) or anti–mouse HRP (Amersham Biosciences, #NA9310) to detect proteins expression. Alternatively, for several western blots we used goat anti-mouse or goat anti-rabbit IgG conjugated with IRDye 680 or IRDye 800 (LI-COR Biotechnology). Immune complexes were detected either by chemiluminescence using Immobilon Western (Millipore, #WBKLS0500) or by the Odyssey Infrared Imaging System (LI-COR Biotechnology). Odyssey v1.2 software (LI-COR) was used for densitometric analysis. OD values were normalized with respect to actin (Millipore AG, #MAB1501) and expressed as a percentage of values obtained for cells infected with control empty vector or cultured at 25 mM glucose (100%).

For immunostaining, 661W cells were cultured as described above and fixed in paraformaldehyde 4% during 45 min prior to be permeabilized and stained for active BAX (Pharmingen, #556467)) and for LAMP2 (Abcam, #ab18528), using FITC Alexa-Fluor 488 goat anti-rabbit antibodies (Invitrogen AG, #A11070) as secondary ones. Cells were counterstained with DAPI (1/1500 in PBS) to identify nuclei and signals were visualized under a fluorescent microscope (Olympus) using appropriate filters.

### Caspase 3 Assay and Immunostaining

Caspase 3 activity was measured with a luminescent Caspase-Glo 3/7 kit (Promega, #G8090) as described by the manufacturer. For the immunostaining of cleaved caspase 3, 661W cells were cultured and fixed as described above and incubated with anti-cleaved Caspase 3 (Cell Signaling Technology, Inc., #9661) and FITC Alexa-Fluor 594 goat anti-rabbit antibody (Invitrogen AG, #A11012) as secondary antibody.

### Immunoprecipitation

Cell lysates used for immunoprecipitation were prepared in CHAPS (3-[(3-Cholamidopropyl)-dimethylammonio]-1-propane sulfonate) buffer (100 mM NaCl, 5 mM NaPO4, 2.5 mM EDTA, 1% CHAPS). Samples containing 300–600 µg of protein were incubated overnight at 4°C with 5 µg of an antibody which recognized the active form of Bax (Pharmingen, #556467). Immunoprecipitates were collected by the addition of protein G Plus-agarose beads (Santa Cruz Biotechnology, Inc., #sc-2002) to each sample followed by incubation overnight at 4°C. Complexes were harvested by centrifugation and washed 4× with PBS prior to be analyzed by western blot using an anti-BAX antibody (Santa Cruz Biotechnology, Inc., #sc-493).

### RT-PCR Analysis

Eight hundred ng of total RNA in a 20 µl reaction mix were used for cDNA synthesis using oligo (dT)_15_ according to the manufacturer’s procedure (First strand cDNA synthesis kit for RT-PCR, Roche Applied Science, #04897030001). The equivalent of 2 to 20 ng original total RNA was used for quantitative PCR amplification using the 2 × brilliant SYBR Green QPCR Master Mix (Stratagene, #600882) and 0.5 µM forward and reverse primer pair, designed to span an intron of the target gene. Real-time PCR was performed in multiple replicates in the Mx3000PTM system (Stratagene) with primers and conditions described in Table S1.

### Electron Microscopy

661W cells were plated in 0.01% poly-L-lysine (Sigma; #P4707)-coated glass slides (LabTek Chamber Slides; Thermo Scientific, #177399) at a density of 1.5 10^4^ cells per slide (area = 1.8 cm^2^), cultured for one day, and finally treated for 48 h with medium containing either normal glucose concentration (25 mM) or low glucose concentration (1 mM). Cells were fixed 2 h in 2.5% glutaraldehyde (Electron Microscopy Sciences, #111-30-8) dissolved in 0.1 M phosphate buffer (PB), pH 7.4. 661W photoreceptor cells were then post-fixed for 1 h in 1% osmium tetroxide (Electron Microscopy Sciences, #QXP010) in PB, and then stained with ethanol 70% containing 1% uranyl acetate (Sigma; #73943). 661W photoreceptor cells were dehydrated in graded alcohol series and embedded in Epon (Sigma; #45345). Ultrathin sections (with silver to gray interference) were cut with a diamond knife (Diatome), mounted on Formvar-coated single slot grids and then counterstained with 3% uranyl acetate and then with lead 0.2% citrate (Sigma; #W302600). Sections were visualized using a Philips CM100 transmission electron microscope.

### Statistical Analysis

All results were expressed as means ± SEM of the indicated number of experiments. Data were statistically analyzed using Prism 6.0 software. We first tested each group of data for distribution normality using Shapiro-Wilk tests. In case of normal distribution, we used a Welch’s ANOVA test (one-way ANOVA with unequal variances) followed by a post-hoc Tukey-Kramer test to compare the different treatments. When the distribution was not normal, we used a Kruskal-Wallis test (non-parametric analogue of the one-way ANOVA) to compare the different treatments. p<0.05 was chosen as the threshold for statistical significance.

## Results

### Low Glucose Induced 661W Photoreceptor Cell Death through Bcl2/Bax Pathway

**Figure 1:**
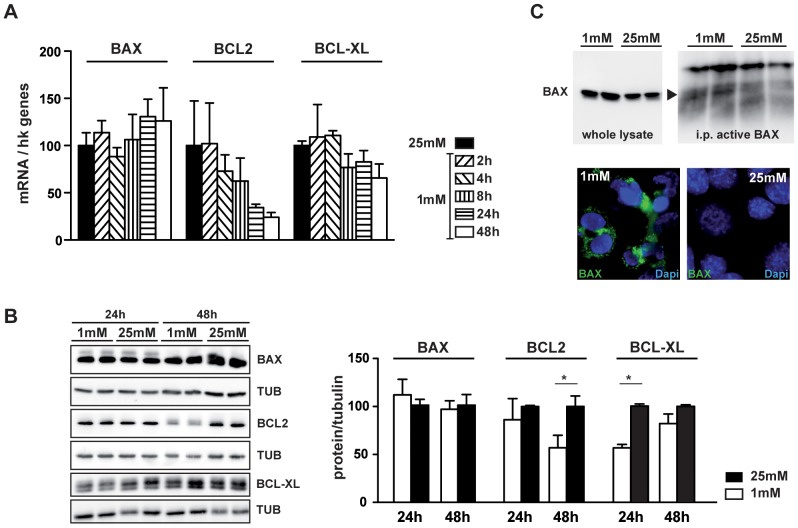
Low glucose induces 661W cell death via the BCL2/BAX pathway. 661W cells were cultured as mentioned in material & methods and then incubated at 1 mM glucose for different time periods (2, 4, 8, 24 and 48 h) or for 48 h at high (25 mM) glucose concentrations. **A)** Graphic representation of *Bcl2*, *Bcl-XL* and *Bax* mRNA expression normalized by housekeeping genes. Results are expressed as mean ± SEM of 2 experiments performed in triplicate. **B)** Western blot of BCL2, BCL-XL and BAX proteins after exposure to 1 mM or 25 mM glucose concentration during 24 or 48 h. Protein amounts were normalized with TUBULIN and compared to 25 mM condition set to 100%. Results are expressed as mean ± SEM of 3 experiments, *p<0.040. **C)** Immunoprecipitation analysis of active BAX from cells exposed to 1 mM or 25 mM glucose during 48 h. Left panel showed the expression of total BAX in whole cell lysates, while right panel showed the expression of immunoprecipitated active BAX (black arrow). Immunofluorescence of active BAX (green) is observed in cells incubated at low glucose during 48 h but not at high glucose conditions.

We first showed that low glucose induced a decrease of *BCL2* mRNA, while *BAX* and *BCL-XL* mRNA expression was not modified (Fig. 1A). We confirmed the decrease of BCL2 at protein level, observing a 45% reduction of the protein content 48 h after low glucose exposure (Fig. 1B). Expression of *BCL-XL*, which was not modified at mRNA levels, was decreased at the protein level after a 24-hour period of culture at low glucose (Fig. 1B). Conversely, BAX protein expression was not changed (Fig. 1B), but BCL2 and BCL-XL decrease leads to the release of active BAX, as showed by immunoprecipitation experiments and confirmed by immunostaining of active BAX in 661W cells exposed to low glucose (Fig. 1C).

### Autophagosomes Accumulation is Induced in 661W Photoreceptor Cells and in Retinal Explants Cultured at Low Glucose

**Figure 2:**
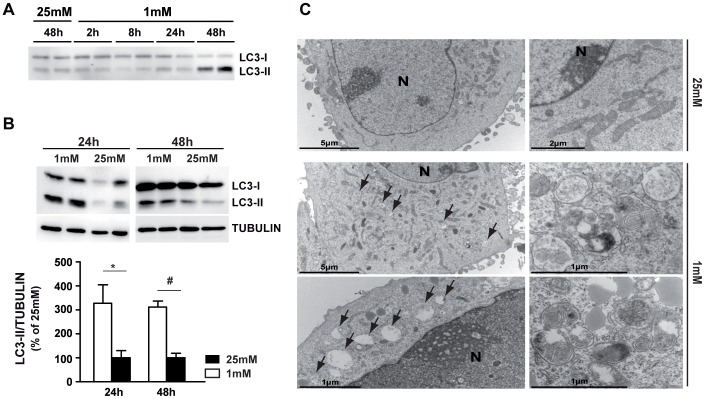
Autophagosomes formation is induced by low glucose in 661W cells and retinal explants. 661W cells and retinal explants were cultured as mentioned in material & methods and then incubated at 1 mM glucose for different time periods (2, 4, 8, 24 and 48 h) or for 48 h at high (25 mM) glucose concentrations. **A)** Western blot of the LC3-II marker of autophagy is representative of 3 different experiments. **B)** Western blot and quantification of LC3-II expression in retinal explants, cultured at 1 mM or 25 mM glucose. Western blot is representative of 2–3 experiments and results are expressed as mean ± SEM of 5 different retinas for each condition, * p<0.03 and # p<0.0001. **C)** Electron micrographs showing the effect of low glucose-induced autophagosome accumulation in 661W photoreceptor cells treated 48 h with 1 mM glucose concentration (arrows) compared to normal glucose concentration (25 mM).

We showed that low glucose conditions increased the expression of the autophagic marker, LC3-II, in 661W cells (Fig. 2A) and in retinal explants (Fig. 2B). We confirmed this result by electron microscopy analysis showing an accumulation of autophagosomes at 1 mM glucose compared to 25 mM (Fig. 2C).

**Figure 3:**
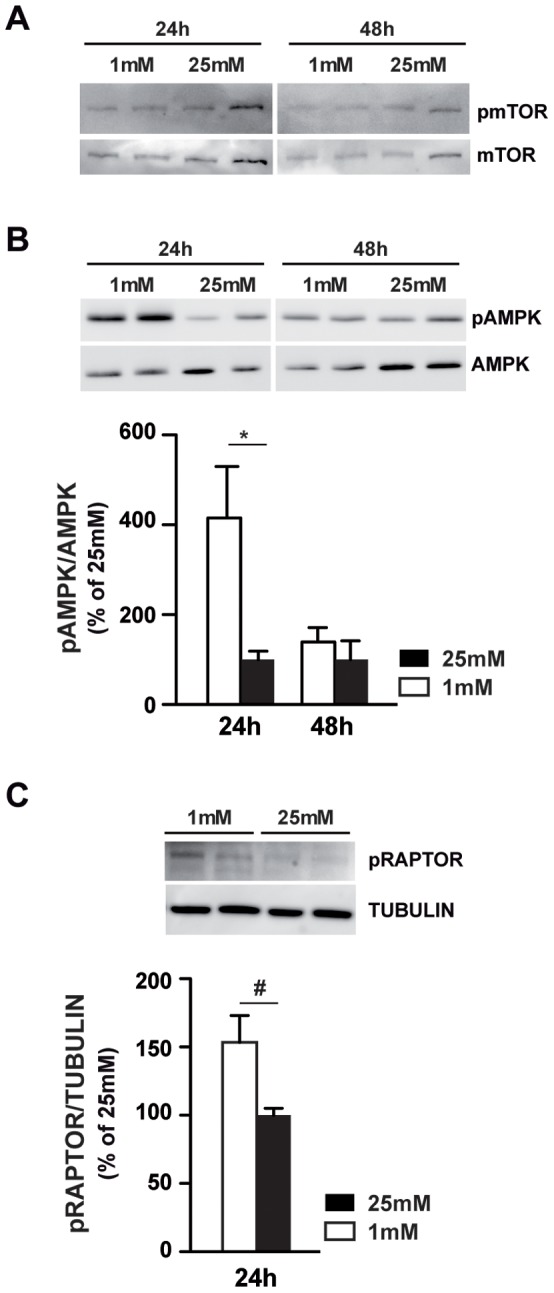
Low glucose induces autophagic flux through AMPK/RAPTOR/mTOR pathway. 661W cells were cultured as mentioned in material & methods, and then incubated at low (1 mM) or high (25 mM) glucose for 24 or 48 h. **A)** Western blot showed the phosphorylation and the expression of mTOR and is representative of three different experiments. **B)** Western blot showed the phosphorylation and the expression of AMPK. Quantifications of pAMPK/AMPK ratio are expressed as mean ± SEM of 3 experiments (n = 8), * p<0.035. **C)** Western blot showed the phosphorylation and the expression of RAPTOR. Oligomycin (Olig) was used at for positive control induction of pRAPTOR. Quantifications of pRAPTOR/TUBULIN ratio are expressed as mean ± SEM of 3 experiments (n = 8), # p<0.019.

To determine the effect of low glucose on autophagic flux and autophagosomes degradation, we tested the regulation of the mTOR pathway. Neither the expression, nor the phosphorylation state of mTOR protein was modified by low glucose (Fig. 3A). As other proteins could modulate mTOR activity, we evaluated the phosphorylation state of AMPK, a nutrient sensor activated by low glucose in different models and implicated in mTOR activity modulation. We were able to show a significant increase of AMPK phosphorylation at low glucose (Fig. 3B). Quantification of the pAMPK/AMPK ratio showed a 4 to 5 fold increase of the phosphorylation form at 24 h and a return to normal values after 48 h. Recently, Gwinn *et al.* showed that AMPK directly regulates mTOR activity by the phosphorylation of mTOR binding partner, RAPTOR. [21] Figure 3C shows that 24 h exposure to low glucose induced the phosphorylation of RAPTOR in 661W photoreceptor cells. Quantification of the pRAPTOR/TUBULIN ratio showed a 1.5 fold increase, which could account for mTOR pathway inhibition and autophagy flux induction. The two anti-apoptotic proteins BCL2 and BCL-XL have also been described to be important factors in autophagy, inhibiting the Beclin1-induced autophagy by binding with BECLIN1 and working together with ATG5 and Ca^2+^ to regulate autophagy. [29] We tested for BECLIN1 mRNA and protein expression, but were unable to observe any change at low glucose (data not shown).

### The Autophagy Flux is Altered by Low Glucose in 661W Photoreceptor Cells

**Figure 4:**
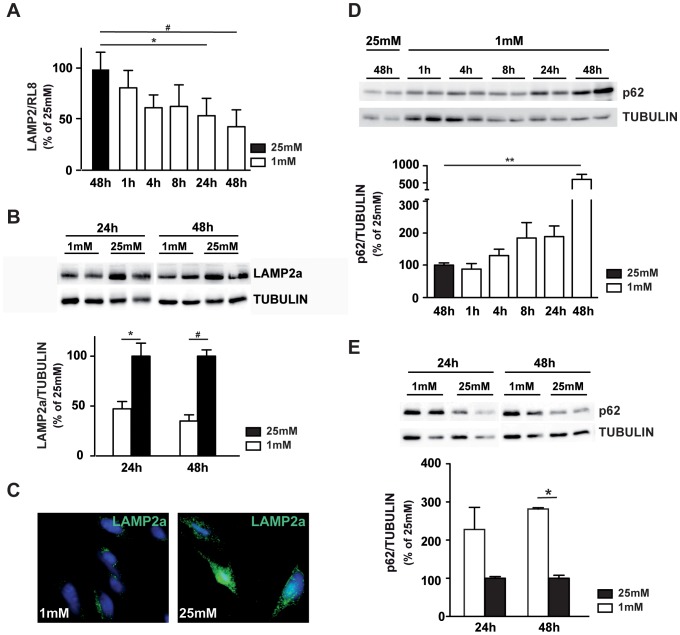
Low glucose induces a decrease in LAMP2 expression. 661W cells were cultured as mentioned in material & methods, and then incubated at low (1 mM) or high (25 mM) glucose for diverse periods of time. **A)** Quantification of *Lamp2* mRNA by qPCR analysis, RL8 gene was used to normalize gene expression. Results are expressed as mean ± SEM of 3 experiments, * p<0.02 and # p<0.0008. **B)** Western blot analysis of LAMP2a protein expression. Results are expressed as mean ± SEM of 3 experiments, * p<0.03 and #p<0.001. **C)** Immunofluorescence staining of LAMP2a (green) in 661W cells cultured at 1 mM and 25 mM glucose concentration. **D)** Western blot analysis and quantification of p62 in 661W cells, western blot is representative of three experiments performed in triplicate and results are expressed as mean ± SEM, ** p<0.0011. **E)** Western blot and quantification of p62 expression in retinal explants, cultured at 1 mM or 25 mM glucose. Western blot is representative of 2–3 experiments and results are expressed as mean ± SEM of 5 different retinas for each condition, * p<0.002.

**Figure 5:**
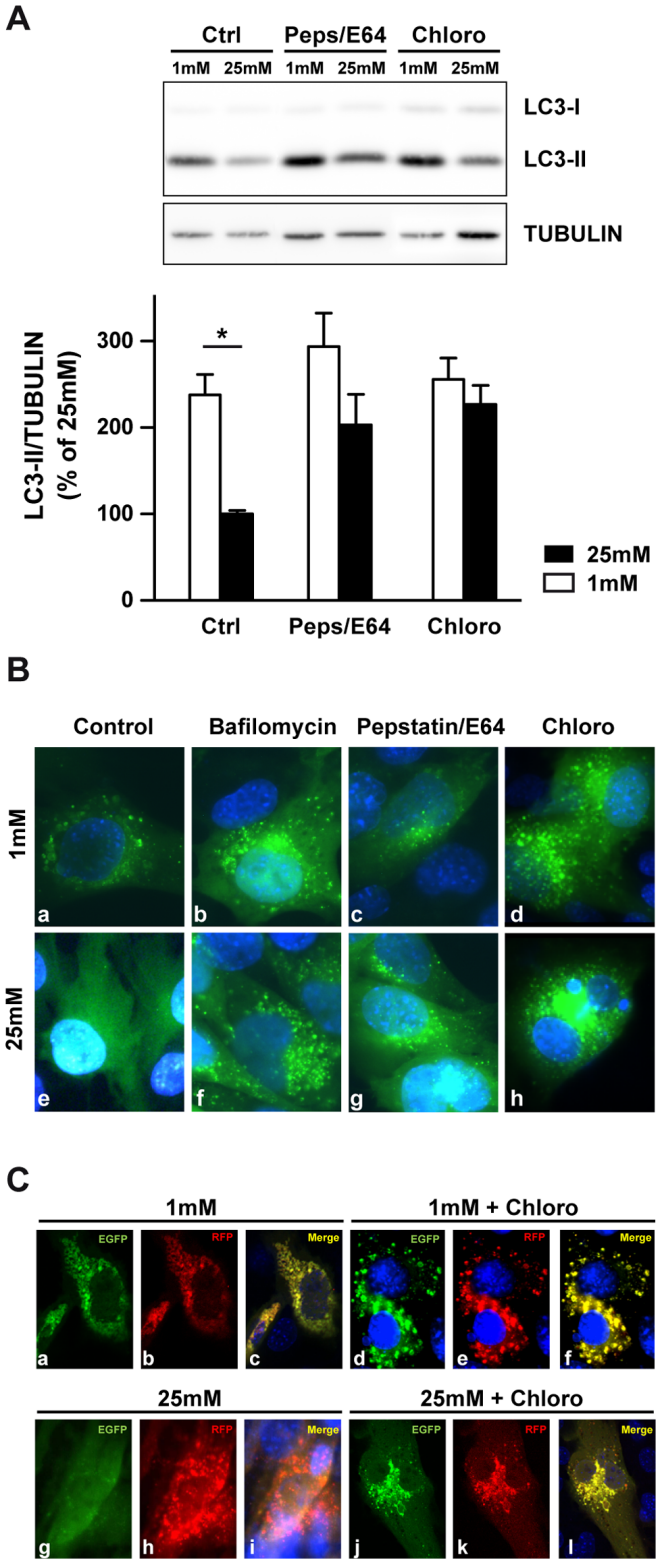
Low glucose-induced LC3-II and p62 accumulation is principally due to a defect of autophagosome/lysosome fusion. 661W cells were cultured as mentioned in material & methods, and then incubated at low (1 mM) or high (25 mM) glucose for 48 h in absence or in presence of fusion inhibitors (10 µg/ml Pepstatin/E64 and 75 µM Chloroquine) during the last 4 h. **A)** Representative western blot analysis of LC3-II protein expression and quantification. Results are expressed as mean ± SEM of 4 experiments, *p<0.03 (1 mM *vs.* 25 mM without inhibitors). **B)** Transfection of 661W cells with a lentivirus expressing the GFP-LC3 chimeric protein and incubation for 48 hrs at 1 mM (a-d) and 25 mM (e-h) glucose in absence (a and e) or in presence of 50 nM Bafilomycin (b and f), 10 µg/ml Pepstatine/E64 (c and g) and 75 µM Chloroquine (d and h) for the last 4 h. **C)** Transfection of 661W cells with a lentivirus vector expressing the mRFP-GFP-LC3 chimeric protein and incubation for 48 h at 1 mM (a–f) and 25 mM (g–l) glucose in absence (a–c and g–i) or in presence (d–f and j–l) of 75 µM chloroquine during the last 4 h.

Our next step was to investigate the expression levels of LAMP2a, a lysosomal protein required for the fusion of lysosomes with autophagosomes [30]. We observed a low glucose-induced time-dependent decrease of *LAMP2* mRNA expression with a significant 50% decrease after 24 and 48 h (Fig. 4A). Western blot analysis (Fig. 4B) and immunofluorescence staining (Fig. 4C) confirmed this result. Accumulation of p62, a protein selectively degraded by autophagy, is also observed in low-glucose cultured 661W cells (Fig. 4D) and in retinal explants (Fig. 4E). To confirm the alteration of fusion process at low glucose conditions, we first used compounds acting as autophagosome/lysosome fusion inhibitors. Pepstatin A coupled with E64 inhibits aspartate and cystein proteases respectively, and chloroquine, a lysosomotropic agent, prevents endosomal/lysosomal acidification. Both treatments prevent the degradation of autophagic cargo inside autolysosomes. Figure 5A shows that inhibitors induced a significant increase of LC3-II at 25 mM glucose, and a slight, but not significant, increased when we compared 1 mM to 25 mM conditions. We then infected 661W cells with lentivirus expressing the GFP-LC3. Figure 5B shows an increase of GFP dot accumulation in all conditions using inhibitors, independent of glucose concentration. This punctuation pattern is characteristic of autophagosomes accumulation. A similar fluorescence pattern was observed at 1 mM glucose without any inhibitor, while attenuation of GFP punctuation was observed at 25 mM glucose. This result suggests either a fusion defect or an increase of autophagic flux. To highlight fusion defect, we infected 661W cells with lentivirus expressing the mRFP-GFP-LC3, which lose its GFP, but not its RFP fluorescence when fusion occurred. We showed that GFP fluorescence was persistent at 1 mM glucose culture condition while no or low GFP fluorescence was observed at 25 mM glucose (Fig. 5C). Meanwhile, RFP fluorescence was similar in both conditions and comparison of merge (RFP and GFP) fluorescence (panels c and i) showed a clear defect of fusion at 1 mM. Interestingly, chloroquine induced similar GFP punctuations at 1 mM and 25 mM glucose (Fig. 5C). These results correlate with LC3-II accumulation (Fig. 5A) and suggested a defect of fusion.

### Autophagy Inhibition Increased Caspase 3 Activity and Cell Death

**Figure 6:**
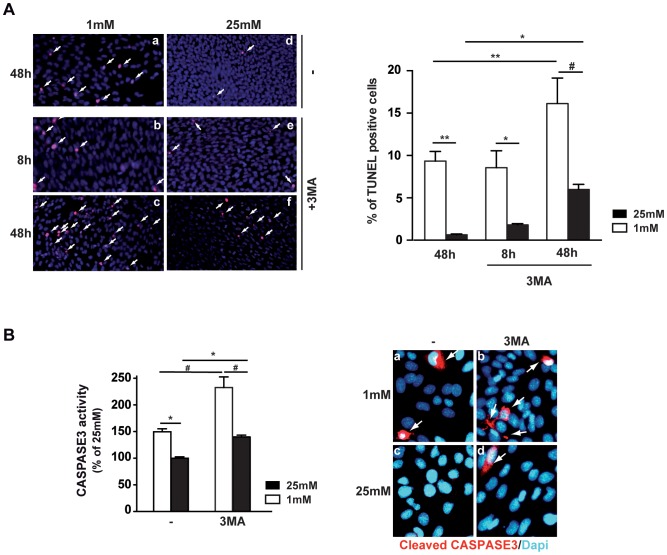
3-MA chemical inhibition of autophagy increases low glucose-induced cell death and caspase 3 activity. 661W cells were cultured as mentioned in material & methods, and then incubated at low (1 mM) or high (25 mM) glucose for different periods of time (8 or 48 h). **A)** TUNEL assay was performed in absence (a, d) or in presence (b, c and e, f) of 600 µM 3-MA, at 1 mM (a, b, c) or 25 mM (d, e, f) glucose concentrations, for different periods of time as indicated to the left. White arrows show TUNEL positive cells. Quantification of TUNEL positive cells was performed in three different experiments, *p<0.05; #p<0.0001 and **p<0.002 **B)** Measures of Caspase 3 activity, results are expressed as mean ± SEM of 3 experiments (n = 13), *p<0.04 and #p<0.0002, and immunostaining of cleaved Caspase 3 in 661W cells incubated at 1 mM (a and b) or 25 mM (c and d) glucose concentrations in absence (a and c) or presence of 600 µM 3-MA (b and d).

Chemical autophagy inhibition, using 3-MA, restores normal levels of low glucose-induced LC3-II expression (Fig. S1) and induced a rapid increase of cell death at both low and normal glucose conditions. Only 8 h after exposure to low glucose, we observed TUNEL positive cells (Fig. 6A, panel b), while no positive cells were observed at this time without 3-MA inhibitor (data not shown). Measurement of caspase 3 activity at both glucose conditions, with or without 3-MA, revealed a significant increase of activity when autophagy was blocked, as shown by caspase 3 assay and immunoreactivity of cleaved caspase 3 (Fig. 6B). Even at high glucose, 3-MA inhibitor induced a significant increase of caspase 3 activity, similar to levels observed at low glucose without the inhibitor.

**Figure 7:**
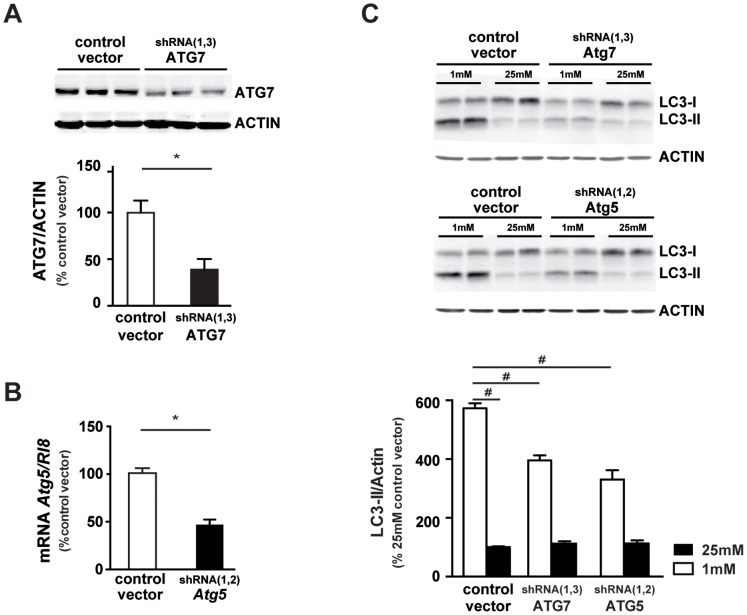
Specific inhibition of ATG5 and ATG7 decrease low glucose-induced LC3-II accumulation. Lentiviruses expressing specific shRNA were used to decrease the expression of ATG5 and ATG7 as described in material & methods, then each clonal cell colony was treated and cultured as mentioned in material & methods. **A)** Representative western blot analysis of ATG7, and quantification. Results are expressed as mean ± SEM of 3 experiments performed in triplicate, *p<0.0001. **B)** Quantification of *Atg5* mRNA expression was performed by PCR and results expressed as mean ± SEM of 2 experiments performed in duplicate, *p<0.002. **C)** Representative western blot analysis of LC3-II expression in specific ATG7 and ATG5 knockdown cells cultured at 1 mM and 25 mM glucose during 48 h and quantification. Results are expressed as mean ± SEM of 3–4 experiments performed in triplicate, #p<0.0001.

**Figure 8:**
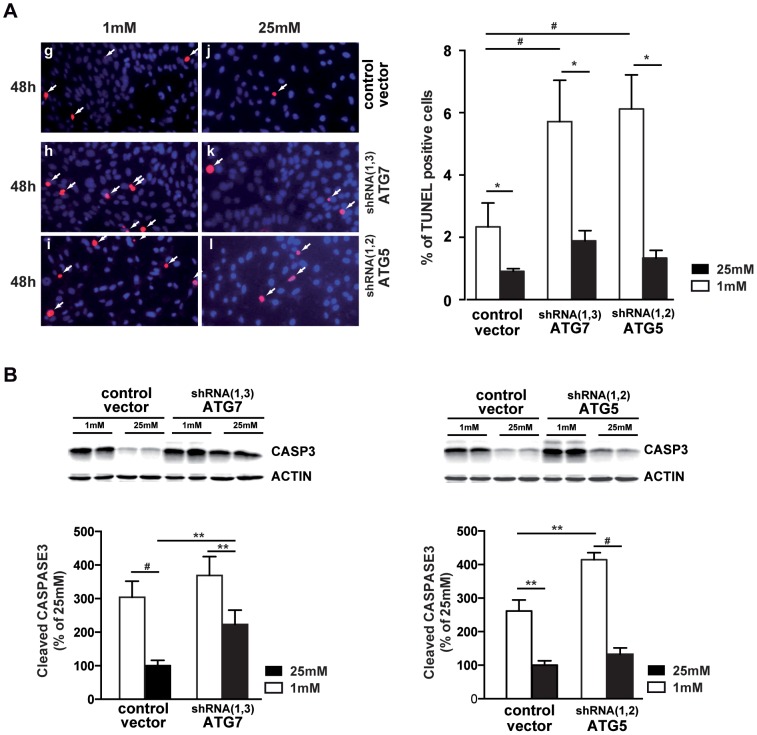
Specific ATG5 or ATG7 inhibition induces cell death and caspase 3 activity. 661W cells were cultured as mentioned in material & methods. **A)** Each clonal cell colony was cultured at 1 mM (g, h, i) or 25 mM (j, k, l) glucose for 48 h prior cell death analysis by TUNEL assay. White arrows show TUNEL positive dying cells and quantification is representative of three distinct experiments, *p<0.005; and #p<0.0001. **B)** Representative western blot and quantification showing cleaved Caspase 3 expression in ATG5 and ATG7 down-regulated clonal cell cultured at 1 mM and 25 mM glucose concentrations. Western blot is representative of three distinct experiments and quantification expressed as 100% of control (25 mM), **p<0.005 and #p<0.0001.

**Figure 9:**
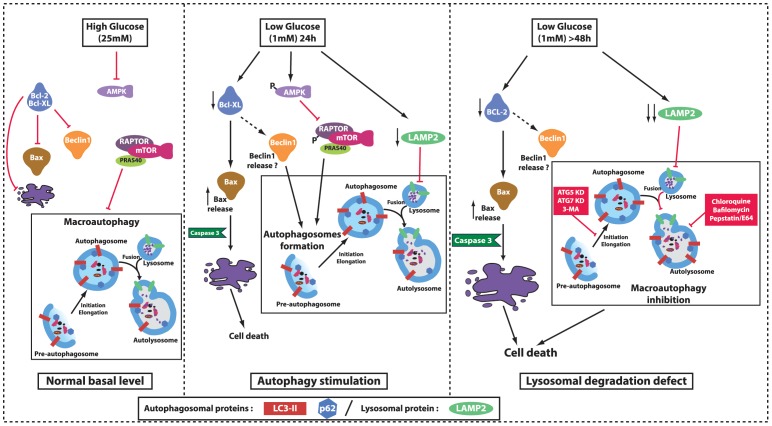
Diagrammatic pathways involved in low glucose-induced autophagy defect. In high (25 mM) glucose concentration, the BCL-2/BAX ratio is not modified, this lead to very low apoptosis; Moreover, AMPK is inactivated and the mTOR complex is able to inhibit autophagosomes formation. On the opposite, in low glucose (1 mM) condition and after 24 h, the autophagic flux is activated via the AMPK/RAPTOR/mTOR pathway. Moreover, low glucose decreases BCL-XL protein expression, which freeing BAX and leads to apoptosis. Direct implication of BCL-2 family proteins in autophagy was not described in this study (dotted lines), but has been described in literature as modulator of free BECLIN1. Longer exposition to low glucose (48 h) induced a decrease in BCL-2 protein expression, which enhanced the effect on BAX (and possibly on BECLIN1). In parallel low glucose induces a decrease in LAMP2 expression, which impaired the autophagosomes/lysosomes fusion process that normally ends autophagy. Inhibition of elongation/maturation process, either chemically (3-MA) or genetically (ATG5/ATG7 KD) led to apoptosis via an increase of Caspase 3. Low glucose. Lysosomal degradation inhibitors (Chloroquine, Bafilomycin and Pepstatin/E64) induce similar pattern than low glucose. Adapted and modified from [21], [51] and [52].

We then down-regulated two autophagic related genes, *Atg7* or *Atg5*, known to play key role in autophagosomes formation. By infecting 661W cells with lentiviruses encoding multiple short hairpin RNAs (shRNAs) against *Atg7* or *Atg5*, we generated specific stable 661W clones. The efficiency of the lentivirus-delivered shRNAs was about 50% for *Atg7* and *Atg5* as shown by Western blot analysis (Fig. 7A) and qPCR (Fig. 7B), respectively. Both down-regulations of ATG7 or ATG5 significantly decreased the low glucose-induced LC3-II expression, indicating that accumulation of autophagosomes was less pronounced in ATG7 and ATG5 shRNAs transduced cells (Fig. 7C). Basal level of LC3-II (at 25 mM) is very low, and modification of low level is difficult to underscore. But figure S2, clearly showed the decrease of Bafilomycin-induced autophagy in ATG5 and ATG7 shRNAs transduced cells, suggesting an inhibition of the autophagic flux in both clonal cells. Down-regulation sensitized cells to low nutrient conditions (Fig. 8A, panels h and i *vs.* panel g). Even at 25 mM, ATG7 or ATG5 down-regulation increased cell death as shown by TUNEL-positive cells (Fig. 8A, panels k and l vs panel j). Quantification showed a significant increase of TUNEL positive cells in ATG7 or ATG5 shRNAs transduced cells. In both clones, the cleaved Caspase 3 was increased at 1 mM glucose conditions in comparison with the control vector (Fig. 8B). Interestingly, ATG7 down-regulation also increased basal level of Caspase 3 activity (Fig. 8B). Apoptosis inhibition, by using the Z-Vad caspase 3 inhibitor [2], tend to slightly decrease the low glucose-induced autophagy (Fig. S1). All together these results, summarized in figure 9, suggest that the inhibition of autophagy sensitized cells to low glucose-induced cell death.

## Discussion

We recently showed that low glucose induces apoptosis in the mouse retina and in 661W photoreceptor cells [2]. In the present study, we showed a decrease of BCL2 and BCL-XL that led to the release of the active pro-apoptotic BAX associated to a failure in autophagic process. In parallel, we showed the low glucose activation of the AMPK/RAPTOR/mTOR pathway in 661W cells [21], [31], which could be responsible for activating the autophagy induction by inhibiting mTOR. This result is in accordance with a recent study showing that Metformin could reduce autophagic vesicles accumulation in pancreatic β-cell of type II diabetes, by restoring normal level of LAMP2 [32]. Interestingly, a recent publication by Wang *et al.* showed a very rapid (10 minutes) activation of AMPK by 2-deoxy-glucose (2-DG), while the LC3-II autophagic marker was only modified after 24 h [22]. Similarly we observed an activation of AMPK only after 24 h and a modification of LC3-II level only after 48 h of 661W cell culture at low glucose. Moreover, the study of Wang *et al.* showed an important role of ROS production in the activation of AMPK and confirmed previous results observed in pancreatic β-cells cultured in glucose deprivation [33]. We also described ROS production in 661W cells cultured at low glucose concentration that could explain the increased activity of AMPK [2].

According to guidelines for study autophagy in eukaryotes [34], [35], we used multiple tools to investigate autophagy and its role in 661W photoreceptor cell death. We first showed an increase of autophagosomes by the increase of LC3-II, the formation of LC3-GFP positive dots and by electron microscopy. This increase could be due to an increase of autophagic flux or to a defect in autophagosomes degradation. Our results using lysosomal inhibitors (Peps/E64) showed a tendency to LC3-II increase at 1 mM in comparison with 25 mM glucose which could suggests an increase in autophagosomes formation 48 h after stimulation. However these observations were not combined with a decrease in p62 levels. One explanation for this result is that induction of autophagy occurs simultaneously with a defect in the autophagosome/lysosome fusion process detected by using a mRFP-GFP-LC3 chimeric protein. We also showed that low glucose induced a progressive reduction of LAMP2a expression 4 h after stimulation. LAMP2a is part of the lysosomal membrane and has been shown to play a key role in the proteolytic pathways, the activity of the chaperone-mediated autophagic (CMA) pathway [36] and autophagosomes maturation [37]. *Lamp2* deficient mice clearly showed an accumulation of autophagic vacuoles [38]. Our results suggested that LAMP2 decrease might play a role in the fusion defect process. It is interesting to note that LAMP2 has been implicated in Danon disease where it caused autophagic vacuolar myopathy in muscles, [39] and retinopathy [40], [41]. A recent publication also described a cone-rod dystrophy, characterized by loss of photoreceptor and RPE cells, in a patient carrying a LAMP2 mutation [42]. Further *in vivo* analysis should provide information about the specific implication of LAMP2 in hypoglycemic-induced retinal cell death and diabetic side effects. This is especially relevant as a recent study associated LAMP2 down-regulation by cholesterol and high fat-rich diet to a decrease in CMA [43].

Interestingly, when we blocked apoptosis with the zVAD inhibitor of caspase 3, we observed reduced autophagy, while autophagy inhibition either chemically or genetically, strongly activated caspase 3 and cell death in low glucose conditions. In our low glucose model, it seems likely that enhanced autophagy represented a survival answer to maintain vital functions of cells, which was counteracted by a lysosomal fusion defect. As autophagy and apoptosis share common pathways (for a review see refs. [18], [19]), we suggest that a crosstalk between these two pathways occurred in 661W photoreceptor cells cultured at low glucose conditions.

Apoptosis was induced by low glucose conditions via modulation of the BCL2/BAX ratio and caspase 3 activation. Proteins of the BCL2 family are also involved in the autophagic process by modulating the level of free BECLIN-1, which is able to induce autophagy [44], [45]. We were unable to see any modification of BECLIN-1 (data not shown), but cannot exclude an increase of free BECLIN-1 due to the observed decrease of BCL2 or BCL-XL proteins. Further analysis is needed to clearly implicate BCL2 family proteins in the low glucose-induced autophagy defect.

We previously demonstrated that, in low glucose conditions, 661W cells produced mitochondrial superoxide [2]. Several years ago, Kunchithapautham & Rohrer (2007) showed a very rapid induction of autophagy (from 1 h) and activation of apoptosis in photoreceptor cells exposed to H_2_O_2_. However in their model of oxidative stress, inhibition of autophagy with 3-MA or ATG5 and BECLIN-1 downregulation decreased caspase-3 activation and cell death [13]. Our opposite results may be explained by the culture conditions, nutrient-rich *vs* deprivation conditions, and by low glucose treatment that induced late production of H_2_O_2_ (24 h) and an autophagosome/lysosome fusion defect.

3-MA compound is not a specific inhibitor of autophagy and could also inhibit class I phosphoinositide 3-kinase (PI3-K) [46], which plays a role in survival pathway by activating the anti-apoptotic and anti-autophagic AKT pathway (also known as protein kinase B (PKB) pathway) [47]. The elevated 3-MA-induced cell death that we observed in 661W cells could then result from the AKT pathway inhibition, as we recorded a 80% decrease of AKT phosphorylation when cells were treated with 3-MA, either at 1 mM or 25 mM glucose (data not shown). Therefore we used a genetic approach, by specifically silencing ATG5 and ATG7 separately, in order to decrease autophagosomes formation; we showed an effect on cell death similar to, albeit to a lesser extent, pharmacological inhibition with 3-MA. All three ways of autophagy inhibition increased 661W cells death.

Recently, several studies suggested an emerging role of autophagy in diabetes mellitus and to organs involved in diabetes side effect [25], [26], [48]. Whether in cardiovascular complications, in diabetic nephropathies or neuropathies, it seems that high ROS concentrations, mostly induced by hyperglycemia, trigger autophagy [25]. Moreover, stimulation of autophagy could improve ER stress-induced diabetes [49], [50]. We showed in our study that hypoglycemia, as well as hyperglycemia, can also be involved in the autophagic process. In conclusion, we hypothesize that autophagy is activated in photoreceptor cells affected by low glucose in order to protect them from apoptosis/necrosis. Unfortunately, low glucose also affects lysosomal degradation or fusion via LAMP2a expression decrease. This leads to a reduced efficiency of autophagic process associated with a strong accumulation of autophagosomes and to an insufficient beneficial supply of energy levels by autophagy. We postulate that pro-autophagic or autophagy stimulating drugs may represent new therapeutic axes to consider in order to reduce certain complications of diabetes such as retinal cells death. The anti-diabetic effect of Metformin (activator of AMPK) might occur partially through the activation of autophagy.

## Supporting Information

Figure S1 **Inhibitor of Caspase 3 slightly affects the low glucose-induced autophagy.** 661W cells were cultured as mentioned in material & methods, and then incubated at low (1 mM) or high (25 mM) glucose for 48 h in absence or in presence of 600 µM 3-MA or Z-Vad. A) Representative western blot analysis and quantification showing LC3-II expression. Results are expressed as mean ± SEM of 2 experiments (n = 4), *p<0.0001. B) Quantification of TUNEL positive cells in presence or in absence of 3-MA inhibitor for 661W cells cultured at low (1 mM) or high (25 mM) glucose concentration. Results are expressed as mean ± SEM of 4 experiments, *p<0.0001 and **p<0.02.
(TIF)

Figure S2 **Specific ATG5 or ATG7 inhibition decreases Bafilomycin-induced LC3-II expression.** Each 661W clonal cell colony was cultured in presence or absence of Bafilomycin (100 nM) for 4 h prior western blot analysis. Empty vector was used as control. Bafilomycin induces an increase of LC3-II, which is decrease when either ATG5 or ATG7 are downregulated in 661W cells. Results are expressed as mean ± SEM of 3 experiments, *p<0.05.
(TIF)

Table S1 **qPCR conditions with primers.**
(TIF)
