# Supplementary figures and images for: Autophagy Defect Is Associated with Low Glucose-Induced Apoptosis in 661W Photoreceptor Cells
Source: PLoS One. 2013 Sep 16;8(9):e74162. doi: 10.1371/journal.pone.0074162 (PMC3774611; doi:10.1371/journal.pone.0074162)

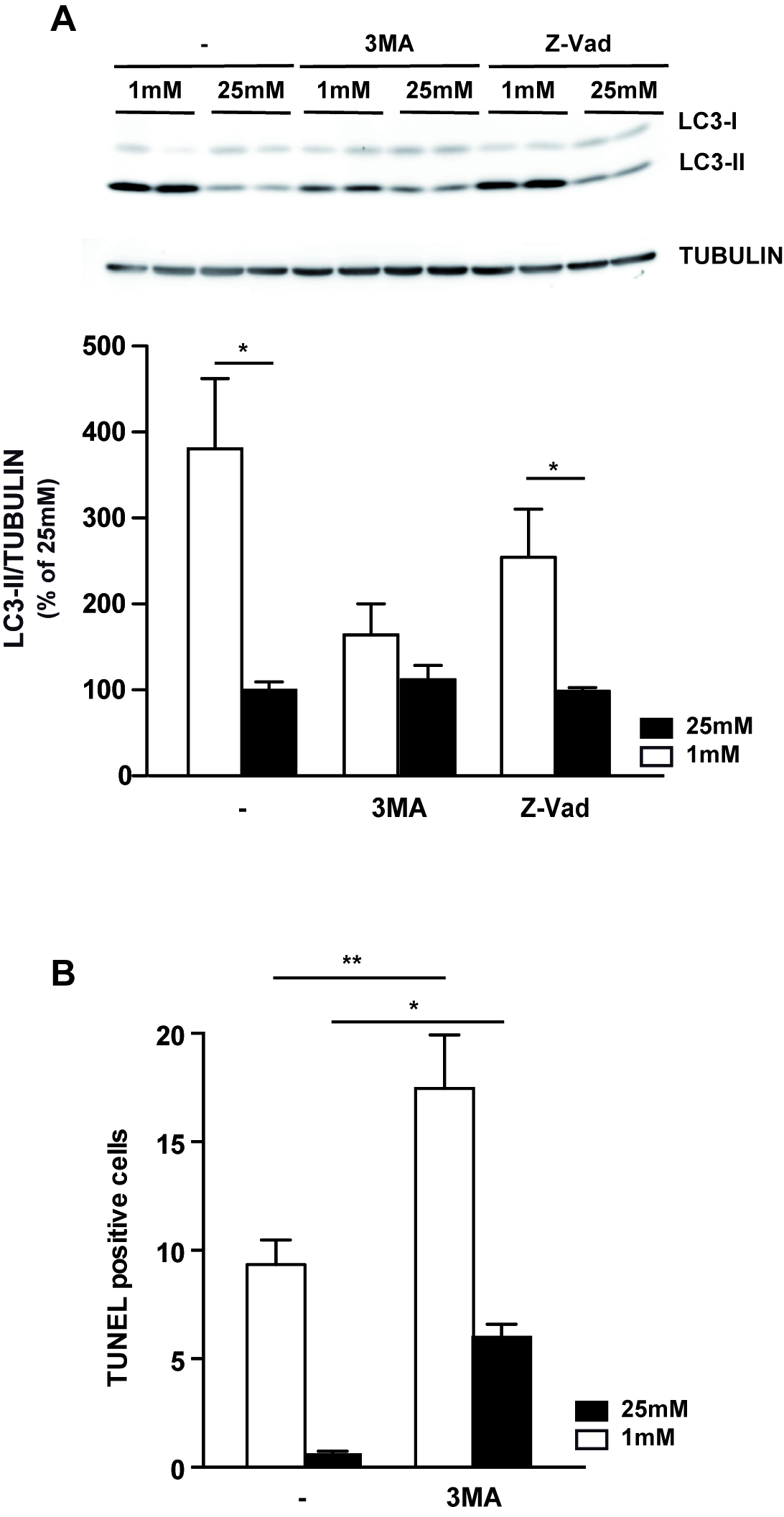

Supplement: Figure S1 — Inhibitor of Caspase 3 slightly affects the low glucose-induced autophagy. 661W cells were cultured as mentioned in material & methods, and then incubated at low (1 mM) or high (25 mM) glucose for 48 h in absence or in presence of 600 µM 3-MA or Z-Vad. A) Representative western blot analysis and quantification showing LC3-II expression. Results are expressed as mean ± SEM of 2 experiments (n = 4), *p<0.0001. B) Quantification of TUNEL positive cells in presence or in absence of 3-MA inhibitor for 661W cells cultured at low (1 mM) or high (25 mM) glucose concentration. Results are expressed as mean ± SEM of 4 experiments, *p<0.0001 and **p<0.02. (TIF) [file pone.0074162.s001.tif]

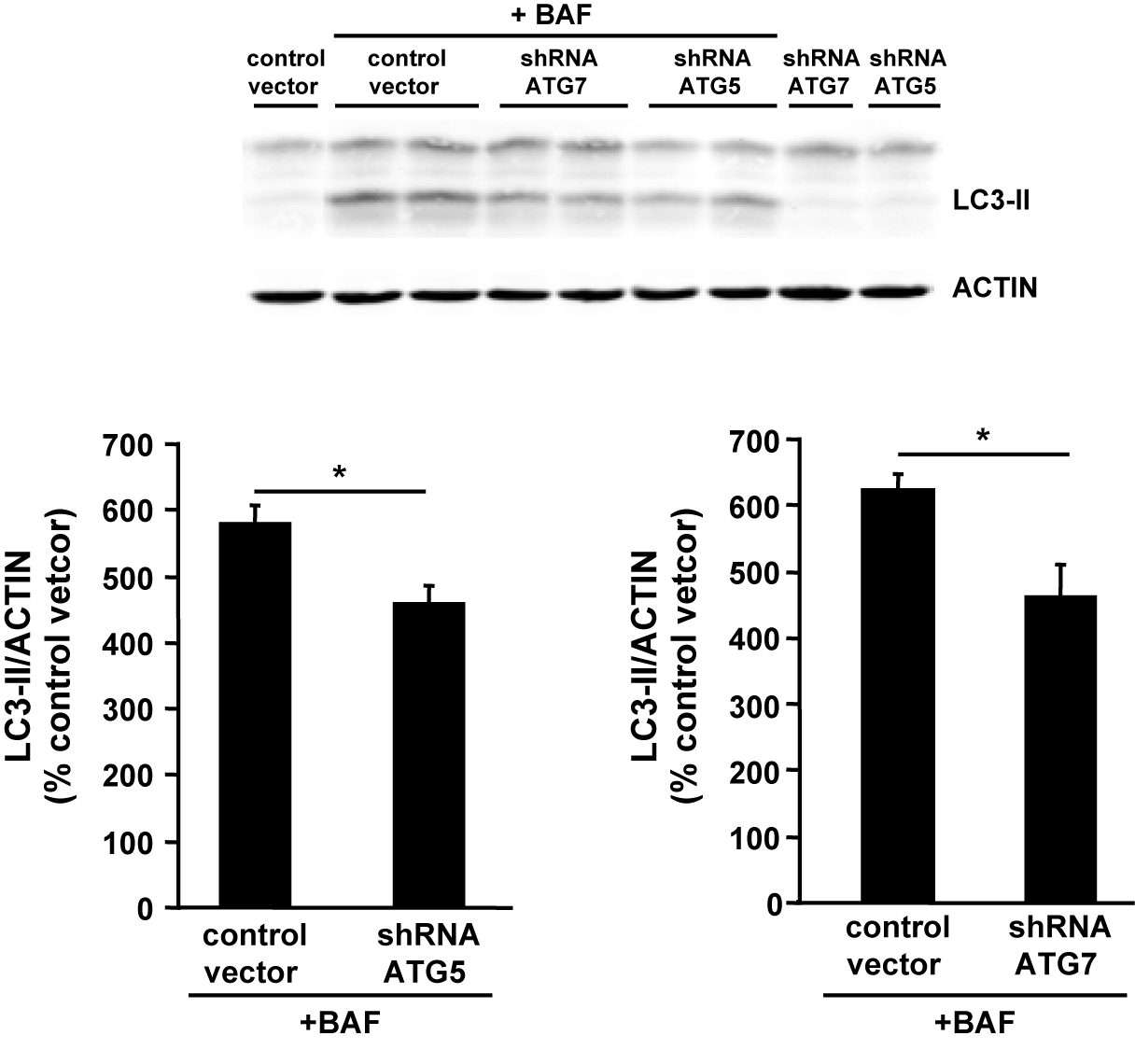

Supplement: Figure S2 — Specific ATG5 or ATG7 inhibition decreases Bafilomycin-induced LC3-II expression. Each 661W clonal cell colony was cultured in presence or absence of Bafilomycin (100 nM) for 4 h prior western blot analysis. Empty vector was used as control. Bafilomycin induces an increase of LC3-II, which is decrease when either ATG5 or ATG7 are downregulated in 661W cells. Results are expressed as mean ± SEM of 3 experiments, *p<0.05. (TIF) [file pone.0074162.s002.tif]

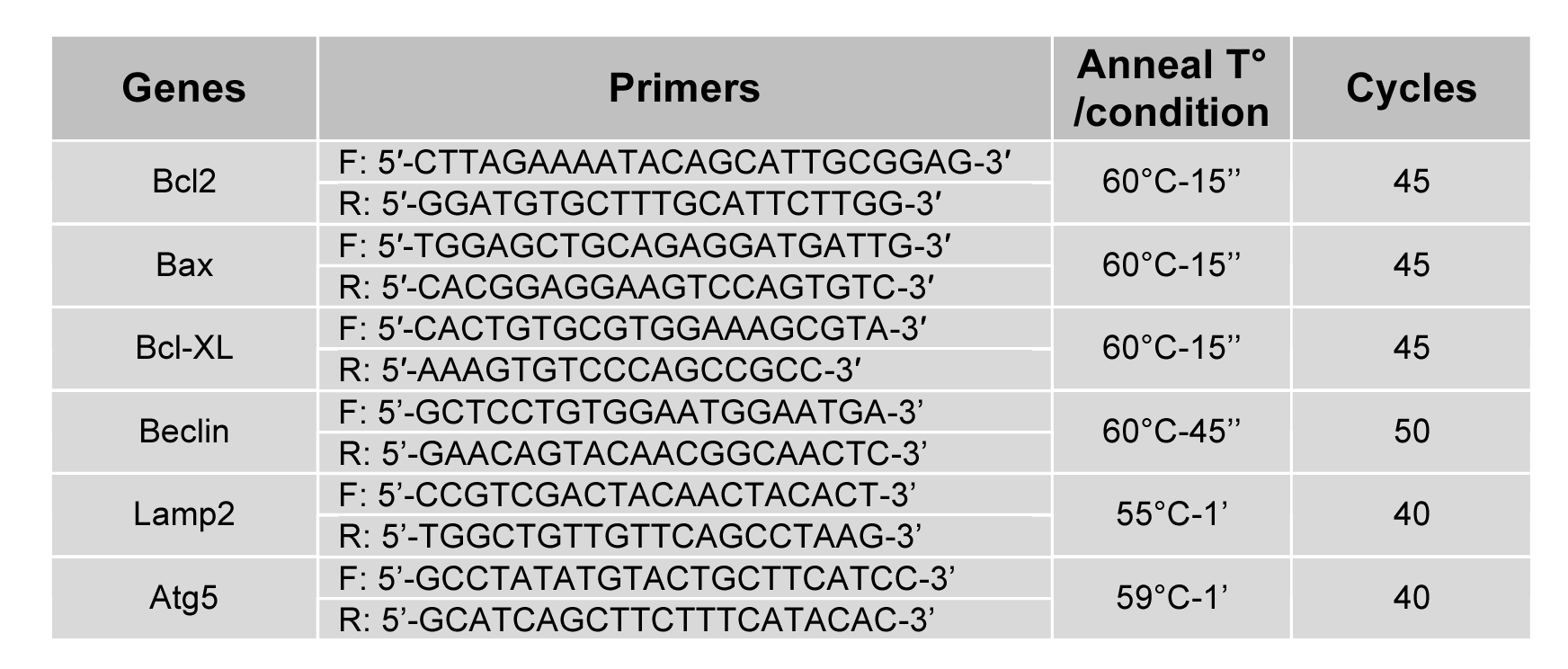

Supplement: Table S1 — qPCR conditions with primers. (TIF) [file pone.0074162.s003.tif]
